# Effectiveness of topical silicone gel and pressure garment therapy for burn scar prevention and management in children: study protocol for a randomised controlled trial

**DOI:** 10.1186/s13063-017-1820-z

**Published:** 2017-02-16

**Authors:** Jodie Wiseman, Megan Simons, Roy Kimble, Robert Ware, Steven McPhail, Zephanie Tyack

**Affiliations:** 1 Centre for Children’s Burns and Trauma Research, Centre for Children’s Health Research, The University of Queensland, Brisbane, QLD Australia; 2 Pegg Leditschke Children’s Burns Centre, Lady Cilento Children’s Hospital, Brisbane, QLD Australia; 3 UQ Child Health Research Centre, The University of Queensland, Brisbane, QLD Australia; 4 School of Public Health and Social Work and Institute of Health and Biomedical Innovation, Queensland University of Technology, Kelvin Grove, QLD Australia; 5 Centre for Functioning and Health Research, Metro South Health, Buranda, QLD 4102 Australia

**Keywords:** Burns, Scar management, Randomised controlled trial, Topical silicone gel, Pressure garment therapy, Children

## Abstract

**Background:**

Abnormal scar development following burn injury can cause substantial physical and psychological distress to children and their families. Common burn scar prevention and management techniques include silicone therapy, pressure garment therapy, or a combination of both.

Currently, no definitive, high-quality evidence is available for the effectiveness of topical silicone gel or pressure garment therapy for the prevention and management of burn scars in the paediatric population. Thus, this study aims to determine the effectiveness of these treatments in children.

**Methods:**

A randomised controlled trial will be conducted at a large tertiary metropolitan children’s hospital in Australia. Participants will be randomised to one of three groups: Strataderm® topical silicone gel only, pressure garment therapy only, or combined Strataderm® topical silicone gel and pressure garment therapy. Participants will include 135 children (45 per group) up to 16 years of age who are referred for scar management for a new burn. Children up to 18 years of age will also be recruited following surgery for burn scar reconstruction. Primary outcomes are scar itch intensity and scar thickness. Secondary outcomes include scar characteristics (e.g. colour, pigmentation, pliability, pain), the patient’s, caregiver’s and therapist’s overall opinion of the scar, health service costs, adherence, health-related quality of life, treatment satisfaction and adverse effects. Measures will be completed on up to two sites per person at baseline and 1 week post scar management commencement, 3 months and 6 months post burn, or post burn scar reconstruction. Data will be analysed using descriptive statistics and univariate and multivariate regression analyses.

**Discussion:**

Results of this study will determine the effectiveness of three noninvasive scar interventions in children at risk of, and with, scarring post burn or post reconstruction.

**Trial registration:**

Australian New Zealand Clinical Trials Registry, ACTRN12616001100482. Registered on 5 August 2016.

**Electronic supplementary material:**

The online version of this article (doi:10.1186/s13063-017-1820-z) contains supplementary material, which is available to authorized users.

## Introduction

The protocol for this study has been reported as per the Standard Protocol Items: Recommendations for Interventional Trial (SPIRIT) guidelines (Additional File 1) [1].

## Background

With post-burn mortality rates declining, the greatest burden to burn centres is scarring [2–4]. In children, scarring has substantial ramifications for the child’s physical and psychological functioning as well as health-related costs for the family and the health care service [5]. Factors associated with risk of scarring in children include total body surface area (TBSA) burned, delayed wound healing, deep burn injuries, skin type, anatomical burn site and skin grafting [2, 6–9].

Abnormal scars have a documented prevalence rate of 32 to 72% post burn [6, 10] and are defined as scars with physical and sensory symptoms that impact on health-related quality of life due to itch, raising, pain, tightness and contracture formation [5, 10]. Qualitative research has also shown that the appearance and the impact of scars must be considered from the patient’s perspective, not just the perspective of the treating clinical team [11, 12]. Scar prevention and management interventions are initiated with the goal of preventing or reducing scar itch, thickness, erythema and pliability with the ultimate goal of maintaining or improving overall appearance of the scar and quality of life [13–18].

Scar thickness has traditionally been one of several characteristics used to define the severity of scarring and is included in most scar rating scales. A meta-analysis of studies of pressure garment therapy effectiveness, a recent systematic review of noninvasive scar interventions, including silicone products and pressure garments and a longitudinal study of scarring in people with burns receiving standard scar management, have supported the importance of measuring scar thickness [2, 17, 19]. Scar thickness has been found to be the characteristic that most clearly distinguishes normal scar and normal skin from hypertrophic scars up to 12 months post burn [19].

Itch has been found post discharge in approximately 80% of patients after burn injury and has been reported to persist for a prolonged period post burn [20, 21]. This symptom has a sustained debilitating impact on patients, influencing wound healing, psychological wellbeing, and engagement in activities of daily living [20, 21]. It is, therefore, important that the prevention and management of burn scars is optimum to reduce the impact of scar sequelae, such as itch, on psychosocial development and health-related quality of life including the child’s ability to independently complete daily activities; and to prevent future invasive scar interventions.

Burn scar prevention and management after skin healing in high-income countries currently includes the use of silicone products or pressure garment therapy or pressure garment therapy combined with silicone products [5]. These treatments have been routine practice for burn scar prevention and management in high-income countries for over 40 years though their effectiveness remains unclear, particularly in children [5, 10, 13].

Silicone products can include topical silicone gels, silicone gel sheets, silicone sprays and silicone oils [22]. A film-forming topical silicone gel was selected for inclusion in this study as topical silicone gels have been recently recommended over other silicone products in clinical practice guidelines as they are thought to result in fewer adverse effects [23]. Strataderm® topical silicone gel, a product used in the participating burns department prior to the trial commencing, has the advantage of a reduced frequency of application (once per day as opposed to twice per day) compared to other topical silicone gel products. It, therefore, has the potential to reduce the treatment burden to patients, improve adherence and reduce the cost of scar prevention and management compared to other scar therapies. Whilst the exact mechanism of action of topical silicone gels has not been confirmed [22–24], it is hypothesised that the occlusive nature of the gel reduces transepidermal water loss, thus increasing the hydration of the stratum corneum [25, 26]. This results in a ‘normalising’ of the cellular processes of the skin, consequently reducing collagen production [25, 26]. Topical silicone gel may also provide a protective barrier against environmental contaminants [25, 26]. It is important to note, however, that whilst there is a reduced risk of adverse effects with the use of topical silicone gels there is still a risk of local dermatological reactions [25].

Pressure garment therapy also works to normalise cellular processes; however, it does this through mechanical pressure. Pressures of 15 to 25 mmHg are hypothesised to reduce capillary flow [13, 18], thus limiting oxygen and nutrients in the affected area and preventing collagen production [27]. The burden of pressure garment therapy to patients and their families, however, can be high if recommended wearing times of 23 h per day for up to 18 months or until scar maturation are followed [2, 28]. Adverse effects from pressure garment therapy can include skin breakdown, altered bone growth and psychological distress from cosmetic differences [2, 5, 7, 29]. The burden of treatment and the presence of adverse effects can impact patient adherence to recommended prevention strategies and requires careful consideration when evaluating the effectiveness of these interventions.

Previous studies have focused solely on the effectiveness of scar management interventions in relation to physical scar characteristics such as itch, height, pain, erythema, and range of motion [17]. They have not considered the broader evaluation of treatment burden (including adverse events), health-related quality of life, or cost-effectiveness [30]. Also, past studies evaluating the effectiveness of topical silicone gels and pressure garments have either not included children, or where children have been included, the numbers have been small or have been case study reports [16, 24, 31]. To overcome the limitations of the evidence in paediatrics, adequately powered, randomised controlled studies that use a broad evaluation framework are required. Thus, a randomised controlled trial (RCT) of the effectiveness of topical silicone gel and pressure garment therapy in children will be conducted using such a framework.

### Objectives

This study aims to determine the effectiveness of topical silicone gel versus pressure garment therapy versus combined topical silicone gel and pressure garment therapy, in the prevention and management of burn scars in children aged 0 to16 years post burn or 0 to18 years post burn scar reconstruction. This will be investigated using the primary outcomes of scar itch and thickness at 6 months post burn or burn scar reconstruction. Current medical literature has been unable to demonstrate one treatment group as being superior to the others; however, we hypothesise that there will be at least one pairwise difference between the group means for scar thickness at the 6-month endpoint.

## Methods

### Study design

**Fig. 1:**
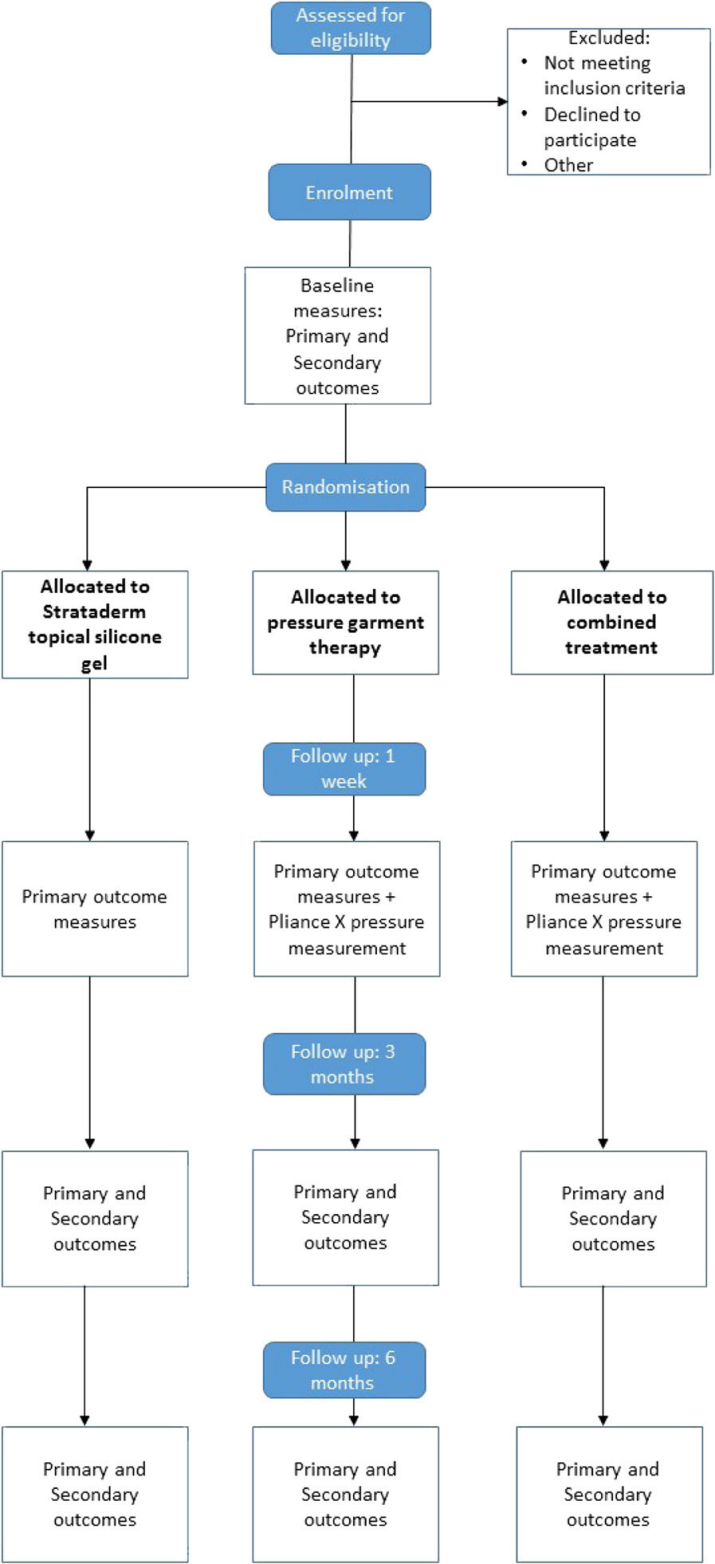
Flow diagram of data collection

A randomised controlled trial with three parallel arms will be conducted to examine the effectiveness of Strataderm® topical silicone gel, pressure garment therapy, and combined topical silicone gel and pressure garment therapy for the prevention and management of burn scars in children. Individual randomisation by patient will be undertaken using computer-generated random numbers. Randomisation will occur using a 1:1:1 ratio between groups in blocks of 12 and will be stratified by surgical intervention received (i.e. skin grafting in the acute phase, spontaneous skin healing in the acute phase, post-acute reconstructive surgery). Concealment of treatment allocation will be completed by the use of sealed, opaque, identical and serially numbered envelopes prepared in advance by an independent party. All outcomes will be measured at baseline (scar intervention commencement), 3 months and 6 months post burn or reconstruction. The primary outcome measures and the level of pressure beneath pressure garments will also be measured at 1 week post baseline. This project has ethics approval from the Child Health Queensland Human Research Ethics Committee (HREC). A flow diagram of the data collection process is displayed in Fig. 1.

A measure of fidelity will assist in determining whether the outcomes are a result of the intervention or the processes involved in delivering the intervention (e.g. the education of participants and caregivers by therapists) [32]. Checklists will be used to record whether the critical elements of the intervention have been delivered as a measure of fidelity. The fidelity checklist includes items regarding mechanism of action of allocated treatment, skin care, sun care, wear and care instructions of allocated treatment and potential adverse effects.

### Setting

The primary setting for data collection will be a burns unit at a large tertiary metropolitan children’s hospital in Australia. Children who access this unit are predominantly male (60%), under the age of 2 years and most frequently present with a scald or a contact burn injury. The incidence of flame and friction burns increases with age [33]. The primary setting provides outreach services via telehealth and receives patients from the Pacific Islands and interstate if required. If necessary, measurements may be taken at a colocated research centre, or the hospital in the patient’s local health district, and/or at the client’s home to reduce potential burden for participants (and their families) and minimise participant dropout.

### Participants

#### Inclusion criteria

The inclusion criteria are children with a burn injury who are managed in the acute phase post burn (up to 16 years of age, as per current practice) or who receive burn scar reconstructive surgery (up to 18 years of age, as per current practice) at the participating burns centre and who require attendance at a scar management clinic after skin healing. Children who receive skin grafting, children with wounds that have not healed by day 17 post burn and children receiving surgery for burn scar reconstruction in all body locations with a TBSA burned of less than or equal to 40% who are accompanied by a parent or guardian, who is able to provide informed consent, will be eligible for inclusion. Up to two scar sites per person will be recruited where possible with both sites receiving the same intervention. Children with a cognitive impairment that impedes their ability to communicate will be enrolled in the study, however, they will not be required to complete self-report measures. Children and their families who do not speak English will be approached to participate in the study with the use of an interpreter but will only have objective scar measures and the itch Numeric Rating Scale (NRS) administered.

#### Exclusion criteria

Exclusion criteria include children whose burns wounds have healed spontaneously within 16 days and who have not been referred for scar management; children with isolated facial or ear burns; children with isolated genital burns; children with comorbidities that might influence the primary outcomes (such as a dermatological disorder); children who are referred to their local health service before scar management is commenced and children involved with the Department of Communities (Child Safety) where the burns consultant and/or burn team members consider study involvement to be contraindicated.

### Intervention

Participants will receive standard care for the acute burn injury as determined by the burns multidisciplinary team. If a participant is recommended for scar management and their caregiver provides consent to participate, baseline assessment will be completed. Participants will then be randomly allocated into one of three scar management intervention groups according to the contents of the sealed envelope opened by an independent party. Group 1 will receive medical use topical silicone gel (Strataderm®). Group 2 will receive pressure garment therapy (Therapeutic Support Laboratory) only. Group 3 will receive a combination of topical silicone gel (Strataderm®) and pressure garment therapy. The intervention products will be provided to the patient as per standard practice once the skin is healed (e.g. sufficient topical silicone gel will be provided to allow coverage of the scar area until the next appointment and two sets of pressure garments will be provided to each child participant at a new fitting).

The study follow-up time periods will match standard occupational therapy review times (e.g. 3 months and 6 months post burn).

### Procedures

It is anticipated that data collection will take approximately 20 min at baseline, 3 and 6 month post burn or burn scar reconstruction appointments. It is expected that data collection will not exceed 10 min at the 1-week follow-up point.

### Outcome measures

#### Primary outcome measures

Scar itch intensity and scar thickness will be measured as primary outcomes. *Itch intensity* will be self-reported for children aged 5 years and older using an 11-point NRS (0 to 10). The Toronto Pediatric Itch Scale is an observation-based scale rating itch behaviours on a scale of 0 (absence of itch) to 4 (severe itch with significant disruption) and will be completed by caregivers for children aged under 5 years [20]. Caregivers for children of all ages will also complete the itch item of the Patient and Observer Scar Assessment Scale (POSAS, 0 to 10 NRS). Numeric rating scales have been recommended over visual analogue scales due to improved adherence, increased responsiveness and fewer missing values in populations of adults with pain and chronic itch [34, 35]. Numerical testing will be conducted with all children aged under 8 years and any children known to have learning difficulties to ensure they have the capacity to use the NRS [36].

*Scar thickness* will be measured using the GE Healthcare ultrasound. The GE Healthcare ultrasound system has been found to have an intraclass correlation coefficient (ICC) of greater than 0.90 for test-retest reliability when used with children with burn scars [37]. When measuring scar thickness with the GE Healthcare ultrasound, an image will be taken centrally from the site of interest (as opposed to peripherally on the scar border). An average of three measurements will be recorded and used in the analysis.

#### Secondary outcome measures

##### Scar severity

Scar severity will be measured using items of the *POSAS* including observer-scale items of thickness, vascularity, pliability, pigmentation and relief and patient scale items of itch, pain, colour, stiffness, thickness and irregularity. The POSAS has been found to be a reliable scar assessment and has the advantage of including a patient-report scale [8]. The POSAS observer scale has been found to have adequate test-retest reliability for pigmentation, pliability and overall opinion of the scar appearance in children [37]. Participants over the age of 8 years, caregivers and therapists will also complete a questionnaire regarding their overall opinion of the scar. The *DSMII ColorMeter* is an objective device that uses tristimulus reflectance colourimetry and narrow-band photometry to measure the lightness, redness or erythema, and pigmentation of a scar [4, 38]. The DSMII ColorMeter has been found to have an interobserver reliability ICC of 0.94 for lightness, 0.94 for erythema and 0.83 for pigmentation when used on scars [38]. At the participating burns centre, the DSMII ColorMeter has been found to have an ICC greater than 0.75 for immediate test-retest reliability on all scales when used with children with burn scars [37].

##### Health-related quality of life

The *Child Health Utility 9D* (CHU9D) (a preference-based utility measure) will be used to measure health-related quality of life and economic evaluation of the interventions. Items covered by the CHU9D include worry, sadness, pain, fatigue, annoyance, school work/homework, sleep, daily routine, and ability to join in activities. Preference weights from these are then converted to quality-adjusted life years for economic evaluation [39]. The CHU9D has been developed with, and validated for, use with children aged 7 to 17 years including validation with an Australian adolescent population [39]. The CHU9D and the *EuroQol 5D - Youth* (EQ5D-Y) have been found to have good levels of agreement (ICC = 0.80) in an Australian adolescent population [40]. Children will self-complete this assessment (from age 5 years) as able and a caregiver proxy report will be completed for children of all ages.

The *Brisbane Burn Scar Impact Profile* will be used to measure the intensity and frequency of sensations, such as pain, tightness and discomfort as well as health-related quality of life specific to people with burn scars. This measure was developed and tested for preliminary validity in children with burn scars and is undergoing further testing with children and caregivers [11]. In adults with burn scars the Brisbane Burn Scar Impact Profile has been found to have acceptable reproducibility, responsiveness and longitudinal validity (Tyack et al. unpublished data).

##### Resource use and costs

*Resource use and costs* will be recorded for each participant from the perspective of the health service provider and costed at market rates. This will include trial interventions costs (e.g. the number and volume of topical silicone gel and pressure garment therapy products), as well as other burn-related resource use (and costs) that may be important to a health service deciding which of the interventions to implement in their model of care for patients with burns (e.g. moulds and splinting, overheads and labour time). Labour time (e.g. occupational therapists, physiotherapist, nurses and surgeons) will be quantified for each patient (on the basis of time duration utilised and number of appointments required) and costed at the relevant state award rates for each respective discipline.

##### Patient adherence

*Patient adherence* to wear and care regimes will be defined as ‘the extent to which a person’s behaviour coincides with medical or health advice’ [41]. Adherence will be measured using self-report questionnaires to child and caregiver participants, which will include questions about the number of hours per day of topical silicone gel and pressure garment therapy wear and reasons for nonadherence to interventions. The percentage of patient-reported nonadherence (using the actual hours of wear and recommended hours of wear) will be measured for each intervention group at the 3- and 6-month follow-up [42]. In addition, adherence will be measured and reported by the numbers of patients randomly assigned to each intervention, receiving the intended treatment, time to cessation of the intended intervention, completing the intended intervention as per the protocol, and analysed for the primary outcomes [42].

##### Treatment satisfaction

Treatment satisfaction has been reported to influence adherence to prescribed medical interventions [43, 44] and so is important to monitor over time. Treatment satisfaction will be measured using a caregiver and treating therapist self-report 0 to 10 NRS.

### Discontinuation/adverse events

The proposed interventions are considered to be part of standard care at the participating burns centre; therefore, minimal adverse events are expected. Known potential adverse events (e.g. a skin rash from topical silicone gel or skin damage from friction caused by pressure garments) have a standardised management protocol at the participating health service. Adverse effects of scar interventions will be monitored by reviewing patient medical records and by the self-report of caregivers, child participants (where appropriate) and treating therapists. All adverse effects will be reported to the clinical health service and the overseeing Human Research Ethics Committee. Discontinuation or alteration of treatment will be at the discretion of the treating clinical team and will be monitored throughout the study. At the conclusion of the study participants will receive standard care.

### Confounding factors

There are a number of potential confounding factors in this study. These include surgical intervention received (i.e. skin grafting in the acute phase, spontaneous skin healing in the acute phase, post-acute reconstructive surgery), patient’s adherence to the treatment regime, whether all essential elements of the intervention were delivered by treating therapists (therapist fidelity), the pressure beneath pressure garments, and/or adverse events impacting on the allocated treatment.

Patient adherence and therapist fidelity will be monitored via self-report checklists of salient elements of the intervention. Therapist fidelity will also be evaluated through the use of audio recordings of 20% of patient sessions [45]. Pressure beneath pressure garments will be measured in millimetres of mercury (mmHg) at the garment skin interface using the Pliance X device [46]. An ICC of 0.998 has been reported for static measures of test-retest reliability and an ICC of 0.995 to 1.00 has been reported for inter-rater reliability of the Pliance X device [47].

### Blinding

Baseline measures will be completed prior to randomisation. Due to the nature of the study double blinding will not be possible as treating therapists and patients and their families will be aware of the treatment modality. Investigator blinding will be difficult to maintain within this environment; therefore, the primary investigator will complete all outcomes measures without blinding. Ultrasound images and the measurement of burn scar thickness using the images taken by the primary investigator will be completed in real time at baseline, 1 week and 3- and 6-month follow-up points. However, at the conclusion of data collection a second investigator (blinded to the results during data collection and to the patient’s identity – nonidentifiable to the second investigator) will measure burn scar thickness using the ultrasound images taken by the primary investigator. Only the results of the blinded assessor will be used in data analysis. The inter-rater variation between the investigators will be reported for the study.

### Statistics

#### Sample size

The sample size estimate was calculated based on the primary outcome of scar thickness at 6 months post burn for one scar site. We assumed a pooled standard deviation of 1.0 mm based on a retrospective audit of data from our centre. To detect a statistically significant between-group difference of 0.76 mm in scar thickness with 80% power and an alpha value of 0.017 (due to three pairwise comparisons), 36 participants are required in each of the three groups. Assuming a 20% dropout, the total number of participants who need to be recruited at baseline is 135 (45 participants per group).

#### Data analysis

Sociodemographic, clinical and intervention data will be summarised using descriptive statistics. For continuous outcomes either mean and standard deviation, or median and range will be used depending on the distribution of the variable. For categorical outcomes frequency and percentage will be reported. Data related to intervention benefits will be analysed on the ‘intention-to-treat’ (ITT) principle, defined as participants analysed according to their randomly allocated study group regardless of treatment received who complete follow-up data collection and who receive the recommended length of intervention. Data related to intervention adverse effects will be analysed according to the ITT principle as well as per protocol defined as those who received and commenced the intervention. A sensitivity analysis will be conducted to compare the ITT and per-protocol approaches if appropriate. Baseline characteristics will be compared to assess the compatibility of groups. At baseline between-group differences will be investigated using linear regression for continuous data and Fisher’s exact test for categorical data. Post-baseline, between-group differences in adherence will also be investigated using linear regression at each time point. Potential confounding variables for the primary outcomes are considered a priori to be: age, days to re-epithelialisation, percentage maximum burn depth, days post burn when scar management commenced, Fitzpatrick skin type, percentage TBSA burned, patient adherence, level of pressure beneath garment (for the groups treated with pressure garments), anatomical body site treated, surgical intervention received (skin grafting in the acute phase, spontaneous wound healing in the acute phase, post-acute reconstructive surgery), wound complications (e.g. infection), other interventions received (e.g. massage) including the use of medications for itch [8, 9, 48].

The primary outcomes will be analysed using mixed effects regression analyses to examine differences at 6 months post burn, adjusting for the baseline value of the outcomes. Mixed effect regression analyses will be completed for each follow-up time point. Potential confounding variables that are identified as imbalanced between the groups will be entered into regression analyses. The time-varying covariates are patient adherence, level of pressure beneath garments, and other interventions received. All of the other potential confounding variables are non-time-dependent covariates. Sensitivity analyses will be completed for missing data using multiple imputation of missing observations by creating ten complete data sets [49] of missing dependent and independent data. For participants from whom data is collected from two scar sites, the multiple sites on these individuals will be accounted for in analytic models by including the scar site as a random effect nested within participant. Initially all participants will be included in the analyses. The analyses will then be repeated after stratifying by surgical intervention (i.e. skin grafting in the acute phase, spontaneous skin healing in the acute phase, post-acute reconstructive surgery). Subsequently, the influence of sociodemographic, clinical and intervention factors, and primary and secondary outcomes not included as dependent variables, on primary and secondary outcomes will also be examined using regression models. Differences in health-related quality of life between the intervention group and respective normative comparison groups will be analysed using *t* tests, z-scores, or equivalent nonparametric tests where appropriate. Differences in self-reported outcomes by children versus caregivers, between age groups and in comparison to normative data will also be examined using univariate statistics such as *t* tests, z-scores, or nonparametric equivalents. Significance will be set at less than 0.05. Data will be analysed using SPSS (SPSS Inc., Chicago, IL, USA) or Stata (StataCorp, College Station, TX, USA) where appropriate.

### Data storage

All information collected as part of this study will be stored safely in a locked filing cabinet in a locked office and in password-protected computer files. Any information obtained in connection with this research project that has the potential to reidentify participants will remain confidential and securely stored. The data collected from this study will be kept for 15 years, as per the recommendations of the National Health and Medical Research Council Guidelines (NHMRC).

## Discussion

Silicone products and pressure garment therapy have been accepted treatments for burn scar management in high-income countries for over 40 years, though there is minimal evidence to support their use [5, 7, 10, 13]. In addition, minimal research has been completed with a paediatric burns population or with any age group using a broad evaluation that investigates the effectiveness of scar prevention interventions, their impact on health-related quality of life, the burden of treatments on patients and their families, or the paediatric patient’s adherence to these interventions.

Whilst little is known about adherence to recommended wear and care regimes for children using topical silicone gel and/or pressure garments, adherence by children is likely less than optimal. In adults, adherence to recommended pressure garment therapy has recently been recorded as 15 to 80% with reasons for nonadherence including the cosmetic appearance of garments, insufficient education regarding garment use as well as poor fit and discomfort [50]. Low rates of adherence to recommended silicone gel sheeting use (24 h per day) have also been reported in adults [51]. It is expected that adherence will be greater when using topical silicone gel (Strataderm®) compared to pressure garment therapy due to its reduced application time and there being no need for additional care/maintenance of the product.

The burden of topical silicone gel also appears lower than pressure garment therapy due to recommended application of the product once or twice daily (depending on scar location), fast drying time and durability. However, topical silicone gels may be time-consuming to apply to an extensive body surface area and have been documented to cause local dermatological reactions [25]. Thus, further investigation of their effectiveness is required.

The burden of pressure garment therapy can be high for patients and their families due to prolonged recommended wearing time, 23 h per day until scar maturation [2, 5], and the need for regular replacement of garments. Pressure garments can also cause discomfort when they are ill-fitting, can instigate changes in bone growth, and can cause heat reactions and friction damage to new skin [2, 7]. In addition to these physical symptoms, pressure garments may also result in emotional and psychological reactions in children with burn scars through visible cosmetic differences, separate from the visible difference that may result from scarring [11].

### Significance of the study

This study will provide evidence of the effectiveness of three burn scar interventions for the prevention and management of burn scars in children. Specifically, evidence will be obtained regarding the effectiveness of topical silicone gel alone, pressure garment therapy alone, and a combination of topical silicone gel and pressure garment therapy. Future randomised control trials will be required to confirm these results; however, if consistent results are achieved, changes in clinical practice may occur.

By using a broad evaluation framework in combination with a design that carefully controls for confounding factors and investigates influencing factors, this study may determine which children benefit most from the scar management interventions of interest. Cost-effectiveness from a health service perspective will also be examined, which, to the knowledge of the authors, has not been examined in scar management intervention studies to date. Examining and controlling for the influence of a range of potential confounders will maximise the rigour of the study and will inform future studies of the factors that influence scar outcomes. Whilst this study will follow patients up to 6 months post burn, it study will pave the way for similar studies with follow-up to scar maturation (which can extend to 18 months post burn or longer) and multicentre trials.

## Trial status

Recruitment commenced at the end of August 2016 and it is expected that recruitment will take approximately 1 year to complete with final data collection occurring in February 2018.

## Additional file

Additional file 1: SPIRIT checklist. (DOC 120 kb)

## Abbreviations

CHU9D: Child Health Utility 9D
EQ5D-Y: EuroQol 5D - Youth
HREC: Human Research Ethics Committee
ICC: Intraclass correlation coefficient
ITT: Intention-to-treat
NHMRC: National Health and Medical Research Council Guidelines
NRS: Numeric rating scale
POSAS: Patient and Observer Scar Assessment Scale
RCT: Randomised controlled trial
TBSA: Total body surface area
